# Omega-3 index and blood pressure responses to eating foods naturally enriched with omega-3 polyunsaturated fatty acids: a randomized controlled trial

**DOI:** 10.1038/s41598-020-71801-5

**Published:** 2020-09-22

**Authors:** Alice V. Stanton, Kirstyn James, Margaret M. Brennan, Fiona O’Donovan, Fahad Buskandar, Kathleen Shortall, Thora El-Sayed, Jean Kennedy, Heather Hayes, Alan G. Fahey, Niall Pender, Simon A. M. Thom, Niamh Moran, David J. Williams, Eamon Dolan

**Affiliations:** 1 Royal College of Surgeons in Ireland, RCSI Education & Research Centre, The Smurfit Building, Beaumont Hospital, Beaumont Road, Dublin 9, DO9 YD60 Ireland; 2 Beaumont Hospital, Dublin, Ireland; 3 Devenish Nutrition, Belfast, UK; 4 University College Dublin, Dublin, Ireland; 5 Trinity College Dublin, Dublin, Ireland; 6 Imperial College, London, UK; 7 Connolly Hospital, Dublin, Ireland

**Keywords:** Biomarkers, Cardiology, Medical research, Risk factors

## Abstract

Diets low in seafood omega-3 polyunsaturated fatty acids (PUFAs) are very prevalent. Such diets have recently been ranked as the sixth most important dietary risk factor—1.5 million deaths and 33 million disability-adjusted life-years worldwide are attributable to this deficiency. Wild oily fish stocks are insufficient to feed the world’s population, and levels of eicosapentaenoic acid and docosahexaenoic acid (DHA) in farmed fish have more than halved in the last 20 years. Here we report on a double-blinded, controlled trial, where 161 healthy normotensive adults were randomly allocated to eat at least three portions/week of omega-3-PUFA enriched (or control) chicken-meat, and to eat at least three omega-3-PUFA enriched (or control) eggs/week, for 6 months. We show that regular consumption of omega-3-PUFA enriched chicken-meat and eggs significantly increased the primary outcome, the red cell omega-3 index (mean difference [98.75% confidence interval] from the group that ate both control foods, 1.7% [0.7, 2.6]). Numbers of subjects with a very high-risk omega-3 index (index < 4%) were more than halved amongst the group that ate both enriched foods. Furthermore, eating the enriched foods resulted in clinically relevant reductions in diastolic blood pressure (− 3.1 mmHg [− 5.8, − 0.3]). We conclude that chicken-meat and eggs, naturally enriched with algae-sourced omega-3-PUFAs, may serve as alternative dietary sources of these essential micronutrients. Unlike many lifestyle interventions, long-term population health benefits do not depend on willingness of individuals to make long-lasting difficult dietary changes, but on the availability of a range of commonly eaten, relatively inexpensive, omega-3-PUFA enriched foods.

## Introduction

Greater consumption of oily fish, and elevated blood levels of the long chain omega-3 polyunsaturated fatty acids (PUFAs), eicosapentaenoic acid (EPA) and docosahexaenoic acid (DHA), are associated with a reduced incidence of heart attacks, arrhythmias, strokes, depression, cognitive decline and Alzheimer’s disease^1–5^. Diets low in seafood omega-3 fatty acids have recently been ranked as the sixth most important dietary risk factor—1.5 million deaths and 33 million disability-adjusted life-years worldwide are attributable to this deficiency^6^.

Because of the strong epidemiological evidence of the benefits of seafood derived omega-3-PUFAs, most national and international guidelines recommend that adults eat at least one serving per week of oily fish (≥ 250 mg/day of EPA and DHA)^7,8^. However, uptake of these recommendations is poor, with many people not consuming any seafood at all. It has been estimated that less than 20% of the world’s population consume the recommended 250 mg of EPA and DHA daily^9^. Consequently, average omega-3-PUFA levels in most countries and regions of the world are sub-optimal^10^. The current main dietary source of EPA and DHA is oily fish. However, globally wild fish stocks are falling, and are insufficient to provide even one portion per week for the world’s population^11^. Aquaculture has steadily grown in recent years, but most farmed fish are dependent on dietary fish oil supplementation from the wild fish catch. This has become a scarce and expensive resource. As a consequence, levels of EPA and DHA in farmed fish have fallen considerably since 2006, such that at least two servings/week of farmed salmon are now required to deliver 250 mg/day of EPA and DHA^12^.

The most recent meta-analyses of randomized controlled trials (RCTs) testing marine omega-3-PUFA supplementation have confirmed protection against cardiovascular morbidity and mortality^13,14^. It is noteworthy that the benefits appear to be most pronounced at very high doses, up to 4,000 mg of long chain omega-3-PUFAs daily^13–15^. Possible explanations for the difference in beneficial dosages of the RCTs compared to the epidemiological studies, include; participant differences—mainly high risk populations, taking multiple cardiovascular medications, were studied in the RCTs, whereas normal populations were in large part studied in the epidemiological studies; poor long-term adherence to supplements; oxidant damage, lessor bioavailability and efficacy of the long-chain polyunsaturated fatty acids when provided as a concentrated fatty acid supplement rather than as a cell membrane phospholipid within a whole food matrix^16^.

The beneficial effects of EPA and DHA in humans are believed to result from their presence in cell membranes. From this location, they influence a myriad of molecular pathways including membrane structure, ion channel properties, genetic regulation, fatty acid metabolism (in hepatocytes), eicosanoid and inflammatory mediator synthesis^17^. It appears likely that some of the beneficial effects of omega-3-PUFAs on cardiovascular health are mediated through downstream effects on blood pressure (BP) and heart rhythm^18,19^.

Hence, in this double-blind, controlled, randomized study, we aimed to quantify the impacts of regular consumption of recently developed alternatives to oily fish, namely chicken-meat and eggs naturally enriched with algae-sourced omega-3-PUFAs, on plasma and tissue (erythrocyte or red cell membrane) levels of DHA and EPA. Furthermore, we tested the hypothesis that the increased blood omega-3-PUFA levels, resulting from regular consumption omega-3-PUFA enriched foods, would have beneficial effects on ambulatory BP levels and heart rate.

## Methods

### Trial design

This was a double-blinded, single-center, randomized controlled trial. Using a 2 × 2 factorial design, 161 healthy community dwelling participants were randomized to eat at least three portions/week of omega-3-PUFA enriched (or control) chicken-meat, and to eat at least three omega-3-PUFA enriched (or control) eggs/week, for 6 months. All methods in this study were performed in accordance with the relevant guidelines and regulations. The protocol was approved by the Beaumont Hospital (Medical Research) Ethics Committee. All participants provided written informed consent. The trial is registered at https://www.clinicaltrials.gov (NCT04127409, 15/10/2019).

### Trial participants

Eligible participants were male or female adults (age ≥ 18 years of age), who were able to provide informed consent. Exclusion criteria included; previous diagnosis of significant cardiovascular disease, such as myocardial infarction, coronary intervention, or stroke; presence of sustained hypertension or dyslipidaemia; concurrent prescription of anti-hypertensive or lipid lowering drugs; and concurrent regular intake of omega-3-PUFA supplements.

### Randomisation and masking

The randomisation schedule was computer generated. Separate sealed opaque envelopes were prepared for male and female participants. Participants and all investigators involved in participant recruitment, data collection and entry, sample collection, processing and analysis, were masked to the randomisation groups until data entry was completed and the database was locked.

### Trial procedures

Participants attended the study center at Beaumont Hospital, at baseline, and at months 3 and 6. At the baseline visit, comprehensive lifestyle and medical histories were taken. A food frequency questionnaire (EPIC-Norfolk)^20^ was completed. Height, weight, and waist circumference were measured, as were office and ambulatory BPs and heart rate. Clinic BP and heart rate were measured using calibrated Omron sphygmomanometers. Ambulatory BP measurements (ABPM) were made every half-hour throughout the 24-h period using SpaceLabs 90207 monitors (SpaceLabs Medical Inc. Issaqua, West Virginia, USA).

Fasting blood samples allowed measurement of plasma and red cell levels of fatty acids, renal function, and plasma lipid levels. All measurements were repeated after 3 and 6 months, and participant well-being, adverse events, concomitant disease activity and the use of any new concomitant medications or supplements were recorded. Participants were asked to avoid taking omega-3-PUFA supplements or anti-hypertensive therapy for the duration of the study.

The poultry meat and eggs were enriched by feeding chickens an algae based feed, which is a rich source of omega-3-PUFAs, particularly DHA. This feed was produced by Devenish Nutrition International (Belfast, Northern Ireland), and supplied to Moy Park Limited (Craigavon, Northern Ireland) and to Skea Eggs (Dungannon, Northern Ireland). As shown in Supplemental Table 1, both the eggs and the chicken-meat were considerably enriched in DHA, but there were only small changes in EPA. Sufficient chilled eggs and frozen chicken-meat (whole chickens, breasts, thighs and wings) were delivered at two weekly intervals to the homes of study participants. This enabled participants, and up to three additional family members, to consume at least three servings per week of chicken meat, and at least three eggs weekly. Participants were encouraged to prepare and cook the chicken-meat and eggs according to their usual practice. Adherence to the assigned diet was assessed at monthly telephone calls or clinic visits—participants were asked how many portions of breast/white and dark chicken-meat, and how many eggs were consumed during the preceding week.

Measurement of plasma and red cell levels of EPA and DHA was performed at the Lipid Analysis Unit, Mylnefield Research Services Ltd, Dundee. Total lipids were extracted from plasma and red cells using a modified Folch method^21,22^. Phospholipids were separated from neutral lipids by 1-dimensional thin-layer chromatography. Fatty acid methyl esters were prepared by direct transesterification and separated using gas chromatography to quantify 28 distinct fatty acid peaks. Identification, precision, and accuracy were continuously evaluated using both model mixtures of known fatty acid methyl esters and established in-house control pools. Interassay coefficients of variation were less than or equal to 5% for both EPA and DHA. Plasma levels of omega-3-PUFAs were expressed in µg/g, and red cell levels as a percentage of total fatty acids in erythrocyte membranes. The erythrocyte or red cell omega-3 index was calculated as the sum of EPA and DHA expressed as percentage of total fatty acids in erythrocyte membranes. Bioavailability of EPA and DHA from omega-3-PUFA enriched foods was calculated as the change in omega-3 index (%) per 100 mg increase in EPA + DHA intake per day. The distribution of omega-3 index was described using the same categories as a recent global survey—< 4% (very low), 4–6% (low), 6–8% (moderate), and > 8% (high)^10^.

### Primary and secondary outcomes

The primary outcome was the change from baseline in the red cell omega-3 index at 6 months. Secondary outcomes included the change in red cell omega-3 index at 3 months, the changes in plasma and erythrocyte levels of EPA and DHA at 3 and 6 months, and the changes in mean 24-h ambulatory BPs and heart rates at 3 and 6 months.

### Statistical analysis

The trial was designed to compare the primary outcome after a follow-up period of 6 months. In a pilot study, 30 healthy community dwelling subjects ate three servings per week of omega-3-PUFA enriched chicken meat for 5 weeks. The omega-3 index increased from 4.93% (standard deviation 1.11%) at baseline to 5.12% (1.18%) after 5 weeks. The mean difference was 0.19% (0.44%). For the current study to achieve a power of 95% to detect a difference of 1% in the omega-3 index between those eating control and enriched chicken-meat, and between those eating control and enriched eggs, at a 2-sided α-level of 0.05, a sample size of 132 participants was required. To compensate for potential losses to follow-up, enrollment of 160 participants was planned for the entire cohort.

Baseline characteristics of the participants are presented as mean and standard deviation (SD) for normally distributed continuous variables, and as median and interquartile range (IQR) for non-normally distributed variables. Dichotomous variables are presented as number and percentage.

Analyses were performed on an intention-to-treat basis on all evaluable participants—those with at least one follow-up measure of the primary outcome. Analysis of variance (ANOVA) was used to compare the changes from baseline after 3 and 6 months of eating the randomly allocated chicken-meat and eggs. Factors included in the ANOVA models were chicken-meat type, egg type and a chicken-meat type*egg type interaction. The groups that ate the enriched eggs, the enriched chicken-meat and both enriched foods were compared to the group that ate both control foods. A fourth comparison was between the groups that ate the enriched eggs and the enriched chicken-meat. P values were adjusted for multiple comparisons using the Bonferroni method. Between group comparisons are presented as mean difference and 98.75% confidence intervals. Sub-group analyses included comparisons of males versus females, older versus younger participants (< 40 years versus ≥ 40 years), and higher versus lower omega-3 index at baseline (< 4% versus ≥ 4%) and higher versus lower baseline BP (mean 24-h BP < 85 mmHg versus ≥ 85 mmHg) levels.

We used SAS 9.4 for statistical analysis.

## Results

### Participants

**Figure 1:**
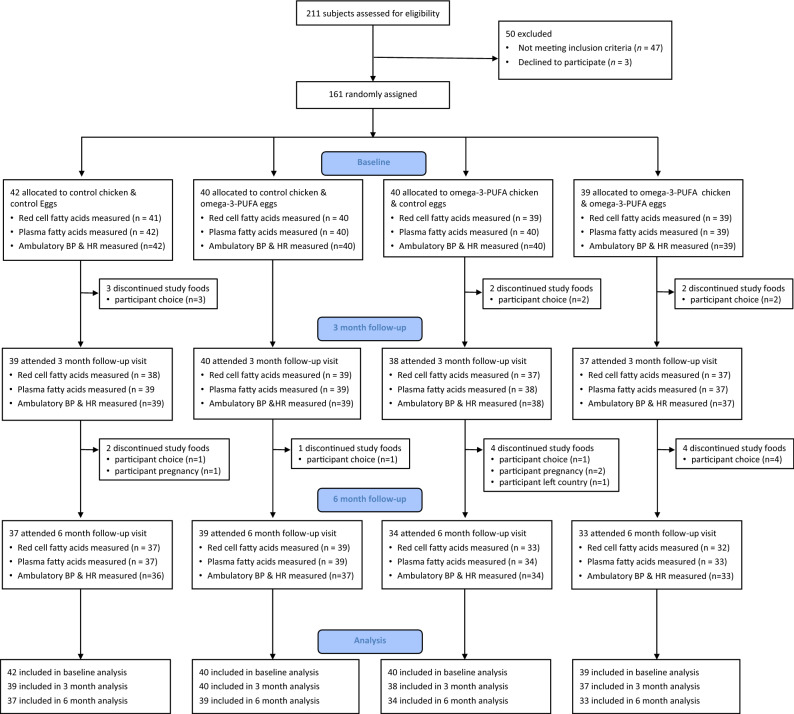
Study consort diagram, illustrating enrolment, intervention allocation, follow-up and data analysis within the trial.

Between February 2015 and January 2016, 211 subjects were assessed for eligibility to the trial (Fig. 1). One hundred and sixty-one met eligibility criteria. Forty-two participants were randomly assigned to eat control chicken-meat and control eggs, 40 to control chicken-meat and omega-3-PUFA eggs, 40 to omega-3-PUFA chicken-meat and control eggs, and 39 to omega-3-PUFA chicken-meat and omega-3-PUFA eggs. Seven participants were lost to follow-up prior to the 3-month visit. Eleven further participants withdrew from the study before the 6-month visit. In total data from 154 and 143 participants were available for the 3- and 6-month follow-up analyses respectively.

Baseline characteristics were well balanced between the four treatment groups (Table 1). Age ranged from 18 to 64 years of age. Seventy-one participants (44%) were male. The majority (99%) were white. Whilst few were current cigarette smokers or drank alcohol to excess, the majority were overweight and did not take sufficient exercise. Forty-two percent of participants regularly consumed one or more portions of oily fish weekly. Both plasma and red cell levels of EPA and DHA were lower than optimal values.

**Table 1:** Baseline characteristics of the intention-to-treat population.

	All subjects (n = 161)	Control chicken and control eggs (n = 42)	Control chicken and omega-3-PUFA eggs (n = 40)	Omega-3-PUFA chicken and control eggs (n = 40)	Omega-3-PUFA chicken and omega-3-PUFA eggs (n = 39)
Age (years)	38.9 ± 10.9	34.9 ± 9.8	40.8 ± 12.1	38.8 ± 9.7	41.4 ± 11.0
Gender (male)	71(44%)	18 (43%)	18 (45%)	19 (48%)	16 (41%)
Ethnicity (caucasian)	159 (99%)	42 (100%)	40 (100%)	38 (95%)	39 (100%)
Current smoker	20 (12%)	5 (12%)	5 (13%)	4 (10%)	6 (15%)
Alcohol use (units/week)	4 [1–10]	3.5 [1–8]	6.5 [2–10.5]	4 [1–12]	3 [0–9.5]
Oily fish intake					
Never/ < 1 portion/month	40 (28%)	12 (32%)	9 (23%)	10 (29%)	9 (26%)
1–3 portions/month	44 (30%)	14 (38%)	9 (23%)	10 (29%)	10 (29%)
1 portion/week	39 (27%)	7 (19%)	13 (33%)	8 (24%)	11 (32%)
2–4 portions/week	18 (12%)	3 (8%)	6 (15%)	6 (18%)	3 (9%)
5–7 portions/week	4 (3%)	1 (3%)	2 (5%)	0 (0%)	1 (3%)
Exercise (30 min sessions/week)	4 [2–6]	3 [2–5]	5 [3–6.5]	3.5 [1–7]	5 [3–6.75]
Body mass index (kg/m^2^)	26.1 ± 5.1	26.5 ± 4.8	25.8 ± 4.8	25.1 ± 3.8	26.9 ± 6.6
Waist circumference (cm)	87.2 ± 12.5	86.8 ± 9.9	87.3 ± 12.5	85.2 ± 10.5	89.9 ± 16.2
Clinic BP and heart rate					
Systolic (mmHg)	118.7 ± 13.8	120.3 ± 11.2	121.5 ± 14.6	117.9 ± 14.1	115.2 ± 14.6
Diastolic (mmHg)	72.0 ± 9.4	74.0 ± 8.1	71.8 ± 10.2	72.1 ± 9.0	70.1 ± 10.2
Heart rate (beats/min)	64.1 ± 9.9	66.2 ± 8.7	62.8 ± 8.5	62.6 ± 10.8	64.8 ± 11.4
Mean 24-h ambulatory BP and heart rate					
Systolic (mmHg)	115.9 ± 8.5	116.4 ± 6.9	116.1 ± 8.2	116.0 ± 9.0	115.2 ± 9.9
Diastolic (mmHg)	68.6 ± 6.4	68.9 ± 7.0	67.7 ± 6.2	69.3 ± 6.8	68.4 ± 5.7
Heart rate (beats/min)	68.0 ± 9.0	69.5 ± 8.0	66.4 ± 9.3	68.0 ± 9.0	69.2 ± 10.5
Fasting lipid levels					
Total cholesterol (mmol/L)	4.97 ± 0.79	4.79 ± 0.86	5.13 ± 0.85	5.09 ± 0.71	4.89 ± 0.70
Triglycerides (mmol/L)	0.96 ± 0.47	0.89 ± 0.45	1.01 ± 0.56	0.95 ± 0.35	0.98 ± 0.49
HDL cholesterol (mmol/L)	1.58 ± 0.36	1.60 ± 0.35	1.60 ± 0.44	1.59 ± 0.31	1.54 ± 0.35
LDL cholesterol (mmol/L)	2.95 ± 0.62	2.78 ± 0.66	3.06 ± 0.63	3.06 ± 0.55	2.91 ± 0.62
Creatinine (micromol/L)	72.5 ± 14.8	72.7 ± 16.8	72.9 ± 13.6	74.9 ± 15.7	69.4 ± 12.7
Plasma fatty acid levels					
EPA + DHA (µg/g)	78.0 ± 31.2	69.0 ± 25.1	83.1 ± 32.9	81.1 ± 35.6	79.3 ± 29.9
EPA (µg/g)	26.8 ± 15.1	22.2 ± 10.4	29.3 ± 16.0	27.8 ± 17.0	28.3 ± 15.8
DHA (µg/g)	51.1 ± 18.7	46.8 ± 16.3	53.7 ± 20.7	53.2 ± 21.4	50.9 ± 15.8
Red cell fatty acid levels (% of fatty acids)					
EPA + DHA (%)	4.52 ± 1.63	4.54 ± 1.47	4.60 ± 1.78	4.61 ± 1.69	4.33 ± 1.60
EPA (%)	0.88 ± 0.42	0.81 ± 0.36	0.93 ± 0.40	0.90 ± 0.44	0.89 ± 0.48
DHA (%)	3.64 ± 1.29	3.73 ± 1.19	3.67 ± 1.44	3.70 ± 1.35	3.44 ± 1.20

Data are n (%), mean ± SD or median [IQR].

*SBP* systolic BP, *DBP* diastolic BP, *HDL* high-density lipoprotein, *LDL* low-density lipoprotein, *EPA* eicosapentaenoic acid, *DHA* docosahexaenoic acid.

### Adherence to allocated diet

Supplementary Table 1 describes the EPA and DHA content of the control and omega-3-PUFA enriched foods. During the study period, according to self-reports, participants ate approximately four portions of chicken-meat and five eggs each week (supplementary Table 2). In those that ate the control foods, the enriched eggs, the enriched chicken-meat and the dual enriched foods, perfect adherence to study instructions of eating at least three servings per week of chicken meat, and at least three eggs weekly, was achieved during 88%, 84%, 85% and 92% of person-weeks, respectively. Estimated total EPA and DHA intake (mean ± standard deviation) from study foods in the four treatment groups was 36 ± 13, 98 ± 43, 91 ± 22 and 156 ± 39 mg/day respectively.

### Effects on the red cell omega-3 index

**Figure 2:**
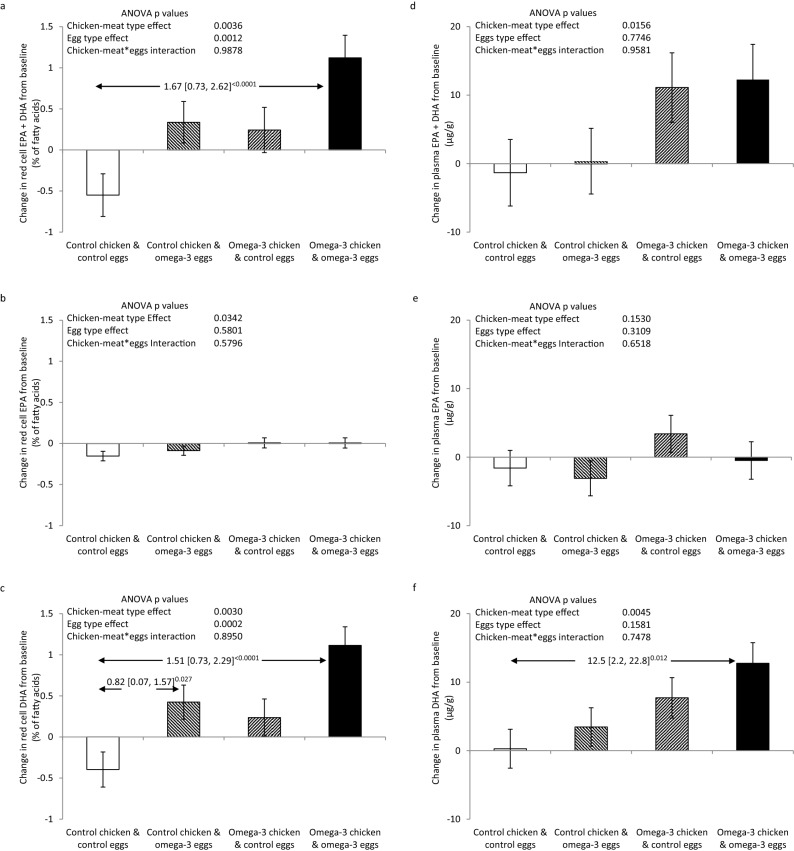
Changes in red cell (**a**–**c**) and plasma (**d**–**f**) levels of EPA and DHA after consumption of control or omega-3-PUFA enriched chicken-meat and eggs for 6 months. Data shown as mean (SEM) change. Statistically significant between group differences are shown as mean difference [98.75 confidence intervals]^Bonferroni adjusted p values^.

After 6 months in the study, the primary outcome, the red cell omega-3 index, increased in those that ate the enriched eggs (p = 0.0012), and in those that ate the enriched chicken-meat (p = 0.0036) (Fig. 2a). It fell over the 6 months in those that ate the control foods − 0.55% (0.26) (mean change (SEM)), but increased in those that ate the enriched eggs (+ 0.34% (0.25)), the enriched chicken-meat (+ 0.24% (0.28)) and both enriched foods (+ 1.12% (0.28)). Absolute differences [98.75% confidence intervals] from the group that ate both control foods were 0.89% [− 0.02, 1.79] with the enriched eggs, 0.79% [− 0.15, 1.74] with the enriched chicken-meat, and 1.67% [0.73, 2.62] with both enriched foods. Figure 2b,c illustrate that the increments in omega-3 index achieved through eating the enriched foods were in large part due to increased DHA levels in the red cell membranes. The changes in EPA levels were small in magnitude, of borderline statistical significance, and were only seen in those eating the enriched chicken-meat. Changes in the omega-3 index and in red cell DHA levels were in large part achieved by the 3 month assessment (supplementary Table 3).

Similar between treatment group differences in the omega-3 index were observed in males and females, in younger and older participants, and in those with lower and higher baseline omega-3 indices (supplementary figure 1). However, female participants and those with a low baseline omega-3 index exhibited smaller reductions with the control foods, and greater increments with the enriched foods—sub-group analyses showed significant gender (p < 0.0001) and baseline omega-3 index (p < 0.0007) effects. These analyses suggest that there may have been some displacement of oily fish from the diets of study participants by the study foods, and that this displacement may have been confined to males.

**Figure 3:**
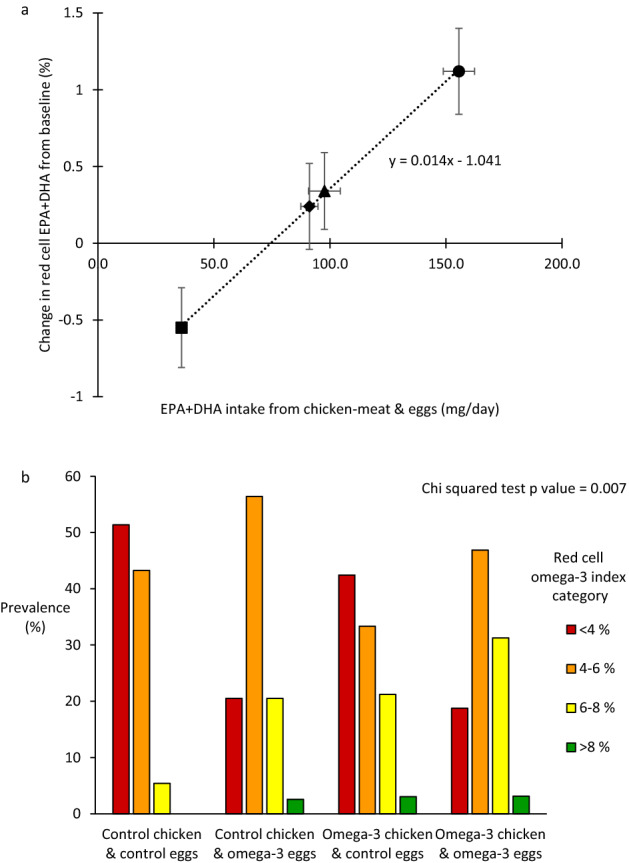
Estimated red cell bioavailability of EPA and DHA, and distribution of red cell omega-3 index after consumption of control or omega-3-PUFA enriched chicken-meat and eggs for 6 months. (**a**) Relationship between the sum of EPA and DHA intake from the study foods, and the change from baseline in the red cell omega-3 index (EPA + DHA % of fatty acids). Data shown as means and SEMs for the groups that ate both control foods (square), the enriched eggs (triangle), the enriched chicken-meat (diamond) and the dual enriched foods (circle). Estimated red cell bioavailability of EPA and DHA is illustrated by the slope of the relationship. (**b**) Distribution of the red cell omega-3 index in the four randomised groups at 6 months. Omega-3 index categories colour coding; red < 4% = very high risk; orange 4–6% = high risk; yellow 6–8% = intermediate risk; and green > 8% = low risk.

There was a linear relationship between the average EPA and DHA intake from study foods in the four treatment groups, and the average change from baseline in the red cell omega-3 index at 6 months (Fig. 3a). Hence red cell (or tissue) bioavailability of EPA and DHA from the two enriched foods separately and in combination was similar—the omega-3 index increased by 1.4% per 100 mg/day of EPA and DHA.

Eating the omega-3-PUFA enriched chicken-meat and eggs for 6 months affected the distribution of the omega-3 index (p = 0.007) (Fig. 3b). By comparison with the group randomised to control foods, those randomised to eating both omega-3-PUFA enriched chicken-meat and eggs had significantly less subjects with a very low omega-3 index (< 4% = very high risk), 18% versus 52%, and considerably more subjects with a moderate or high omega-3 index (> 6% = intermediate or low risk), 38% versus 6%.

### Effects on plasma omega-3-PUFA levels

Changes in plasma levels of EPA and DHA at 3 and 6 months were similar to the red cell changes (Fig. 2d–f, and supplementary table 3). However, as expected, greater variation was evident for plasma versus red cell measures, and generally, the differences were not as statistically significant.

### Effects on BP and heart rate

**Figure 4:**
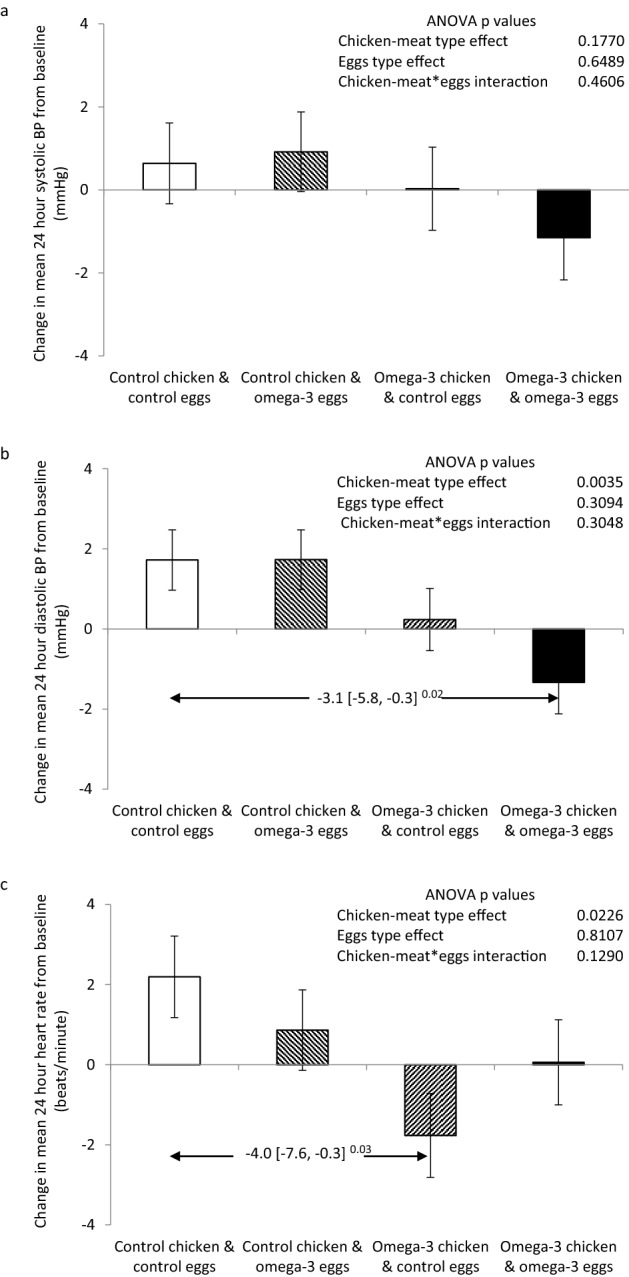
Changes in mean 24-h ambulatory systolic and diastolic blood pressure (BP) (**a**,**b**) and heart rate (**c**), after consumption of control or omega-3-PUFA enriched chicken-meat and eggs for 6 months. Data shown as mean (SEM) change. Statistically significant between-group differences are shown as mean difference [98.75 confidence intervals]^Bonferroni adjusted p values^.

Figure 4 illustrates the effects of the enriched foods on BP and heart rate. There was a non-significant trend for mean 24-h systolic BP to increase with the control foods, and to decrease with the enriched foods. Mean 24-h diastolic BP was reduced by eating the enriched chicken-meat (p = 0.0035). The absolute difference between the control group and the dual enriched foods group was − 3.1 mmHg [− 5.8, − 0.3]^0.02^. Sub-group analysis showed a significant baseline BP effect on the changes in diastolic BP (p = 0.0013) (supplementary figure 2). While between treatment group differences were similar to the whole group, participants with a low baseline BP (mean 24-h BP < 85 mmHg) exhibited a larger increment with the control foods, and an unchanged pressure with the enriched foods, whereas participants whose baseline mean 24-h BP was greater than 85 mmHg, exhibited no change in diastolic pressure with the control foods, and a larger reduction with the enriched foods. Sustained hypertension (baseline daytime BP greater than or equal to 140/90 mmHg) was an exclusion criterion for this study. Hence, both the decrease in the omega-3 index, and regression to the mean, likely contributed to the observed rise in diastolic BP in the group that ate both control foods.

Similar to BP levels, mean 24-h heart rate appeared to rise with the control foods but declined with the enriched chicken-meat (p = 0.0226) (Fig. 4c). The absolute difference from the group that ate both control foods was greatest for the group that ate the enriched chicken-meat and control eggs (− 4.0 beats/min [− 7.6, − 0.3]).

## Discussion

Regular consumption of omega-3-PUFA enriched chicken-meat and eggs significantly increased red cell levels of essential long-chain omega-3-PUFAs in community dwelling healthy volunteer participants. Bioavailability of EPA and DHA from the two types of enriched foods was similar and excellent. Eating both enriched foods beneficially altered the distribution of the omega-3 index—there was a more than halving in the number of subjects with a high-risk omega-3 index. In addition, eating the enriched foods resulted in clinically relevant reductions in diastolic BP and heart rate—both established biomarkers of cardiovascular health.

In our trial, participants allocated to eat both enriched foods had an additional daily intake of EPA and DHA of approximately 120 mg. A recent systematic review of dietary risks indicates that mortality risk decreases by 10–30% with each 100 mg increment in dietary EPA and DHA intake^6^. By comparison with the group that ate both control foods, the red cell omega-3 index increased by 1.7% in those that ate both enriched foods for 6 months. We chose the red cell omega-3 index as the primary outcome, because of its lessor variability over time compared to plasma values^23,24^, and because of its proven correlation with EPA and DHA levels in human cardiac tissue^25^, and in multiple relevant organs in animal models^26^. Importantly the omega-3 index has been shown to predict cardiovascular events, and all-cause mortality^27–29^. In a recently completed Framingham study, each 1% increment in the omega-3 index was associated with a 15% decrement in cardiovascular events and a 10% decrement in total mortality^29^.

The observed decreases in 24-h ambulatory diastolic pressures and heart rate with the enriched foods support the hypothesis that the increased dietary intake of long-chain omega-3-PUFAs from the enriched foods, and the resultant elevated tissue levels, truly provide cardiovascular protection. Ambulatory measurements of BP have been shown to be superior to clinic measurements in predicting cardiovascular morbidity and mortality^30–32^. An analysis of data from the Spanish Ambulatory BP Registry suggests a 50% increase in all cause and cardiovascular mortality for every 10 mmHg increase in population 24-h ambulatory diastolic BP^32^. Hence, the 3 mmHg difference observed in our trial, could translate into important whole population reductions in cardiovascular morbidity and mortality.

It has been estimated that less than 20% of the world’s population consumes the recommended minimum of 250 mg/day of long-chain omega-3-PUFAs (EPA and DHA) ^9^. As a consequence it is not surprising that few regions, principally Japan, Scandinavia and areas with indigenous populations not fully adapted to westernized food habits, demonstrate optimal blood levels (omega-3 index ≥ 8%)^10^. Very low levels, an index of < 4% have been observed throughout North America, central and South America, Europe, the Middle East, Southeast Asia, and Africa^10^. Baseline omega-3 index in our study population, at 4.5%, is in concordance with this global survey.

Several strengths and limitations merit careful consideration. The diets of the participants were neither tightly controlled nor supervised. Indeed participants were only asked to eat at least three portions of chicken-meat and at least three eggs per week—they were provided with considerably more study foods than this minimum, and were free to eat more than the required three portions of each. Consequently, within each treatment group, there was considerable variation in EPA and DHA intake. Despite this variability, significant differences in the primary and secondary outcomes were detected. Indeed the change in diastolic BP recorded in our study was of a similar magnitude to that recorded amongst normotensive participants, in response to the very stringently supervised diets, of the Dietary Approaches to Stop Hypertension (DASH) trials^33,34^. We believe that the generalizability of the results of our trial are enhanced by this realistic approach.

We acknowledge that the amount of omega-3-PUFAs delivered within a portion of chicken-meat or an egg is relatively small in comparison to that potentially contained within a portion of oily fish or a supplement. However, the excellent bioavailability of EPA and DHA from the study foods in part compensates for this. The calculated red cell bioavailability of EPA and DHA from the omega-3-PUFA enriched chicken-meat and eggs was 1.4% per 100 mg/day of EPA and DHA. This appears to equal or exceed that previously recorded for oily fish (0.6–0.8%), and fish oil supplements (0.2–0.6%)^35,36^. Furthermore, adherence to the allocated diets within the study was excellent, and compares favorably with other lifestyle and pharmacological strategies for the primary prevention of cardiovascular events^37^. Hence, the enriched foods are likely to be frequently eaten by large numbers of people, including children, facilitating consistent lifelong adherence.

In conclusion, improved omega-3-PUFA levels in humans is likely to lead to substantial human health benefits, including protection from heart attacks, strokes, dementia, and depression. Omega-3-PUFA enriched chicken-meat and eggs offers consumers attractive additional alternatives to eating oily fish. Unlike many lifestyle interventions, long-term population health benefits will not be dependent on individual willingness to make long-lasting difficult dietary changes, but on the availability of a range of commonly eaten and inexpensive foods, that have been naturally enriched with omega-3-PUFAs.

## Supplementary information

Supplementary Information 1.

## Data Availability

The full trial protocol and the de-identified datasets that were analyzed during the current study will be made available by the corresponding author upon reasonable request.
