# Supplementary material for: Omega-3 index and blood pressure responses to eating foods naturally enriched with omega-3 polyunsaturated fatty acids: a randomized controlled trial
Source: Sci Rep. 2020 Sep 22;10:15444. doi: 10.1038/s41598-020-71801-5 (PMC7508802; doi:10.1038/s41598-020-71801-5)
Supplement: Supplementary file 1 — Supplementary Information 1. [file 41598_2020_71801_MOESM1_ESM.docx]

**Supplementary Information**

**Omega-3 index and blood pressure responses to eating foods naturally enriched with omega-3 polyunsaturated fatty acids; a randomized controlled trial**

Alice V. Stanton, M.B., Ph.D.,^1,2,3^ Kirstyn James, M.B., M.D.,^1,2^ Margaret M. Brennan, M.B.^1^ Fiona O’Donovan, B.A., M.Sc.^1,3^ Fahad Buskandar, M.B.,^1^ Kathleen Shortall, B.S.N.,^1^ Thora El-Sayed, B.S.N.,^1^ Jean Kennedy, Ph.D.,^3^ Heather Hayes, Ph.D.,^3^ Alan G. Fahey, Ph.D.,^4^ Niall Pender, Ph.D.,^1,2,5^ Simon A.M. Thom, M.B., M.D.,^6^ Niamh Moran, Ph.D.,^1^ David J. Williams, M.B., Ph.D.,^1,2^ and Eamon Dolan, M.B., M.D..^1,7^

1. Royal College of Surgeons in Ireland, Dublin, Ireland.
2. Beaumont Hospital, Dublin, Ireland.
3. Devenish Nutrition, Belfast, United Kingdom,
4. University College Dublin, Dublin, Ireland.
5. Trinity College Dublin, Ireland.
6. Imperial College, London, United Kingdom.
7. Connolly Hospital, Dublin, Ireland.

**Corresponding Author:**

Professor Alice V. Stanton,

Royal College of Surgeons in Ireland, RCSI Education & Research Centre, The Smurfit Building, Beaumont Hospital, Beaumont Road, Dublin 9, DO9 YD60, Ireland

Telephone: +353 1 809 3735

Email: astanton@rcsi.ie

**Contents**

Page

Supplementary figure 1 3

Supplementary figure 2 4

Supplementary table 1 5

Supplementary table 2 6

Supplementary table 3 7 & 8

Supplementary table 4 9

**Supplemental Figure 1:** Changes in the red cell omega-3 index from baseline after consumption of control or omega-3 PUFA enriched chicken-meat and eggs for 6 months, in females and males (panel A), in younger and older subjects (panel B), in those with lower and higher omega-3 indices at baseline (panel C), and in those with lower and higher baseline blood pressures (panel D). Data shown as mean (SEM) change.

**
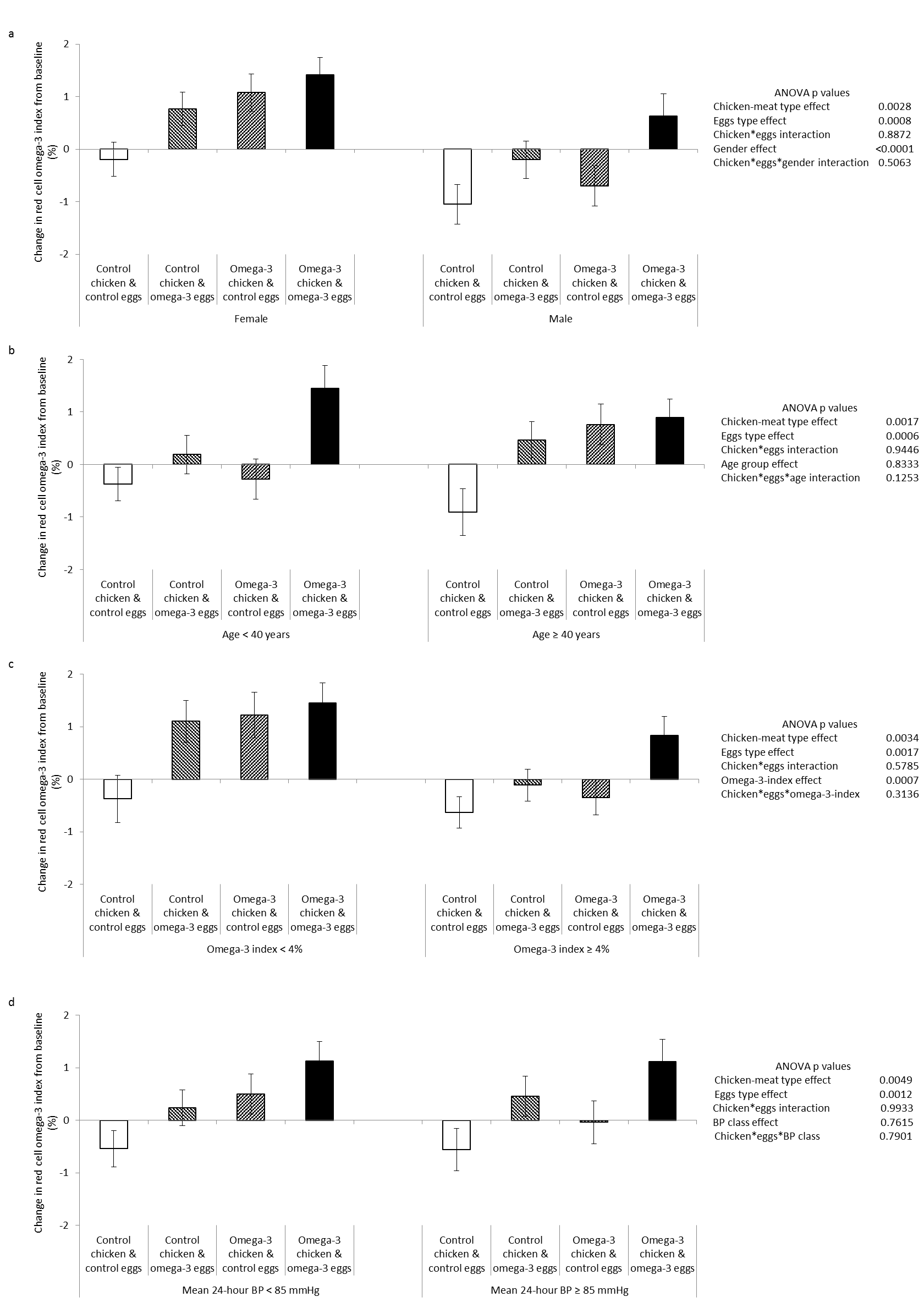
**

**Supplemental Figure 2:** Changes in mean 24-hour ambulatory diastolic BP from baseline after consumption of control or omega-3 PUFA enriched chicken-meat and eggs for 6 months, in females and males (panel A), in younger and older subjects (panel B), in those with lower and higher omega-3 indices at baseline (panel C), and in those with lower and higher baseline blood pressures (panel D). Data shown as mean (SEM) change.

**
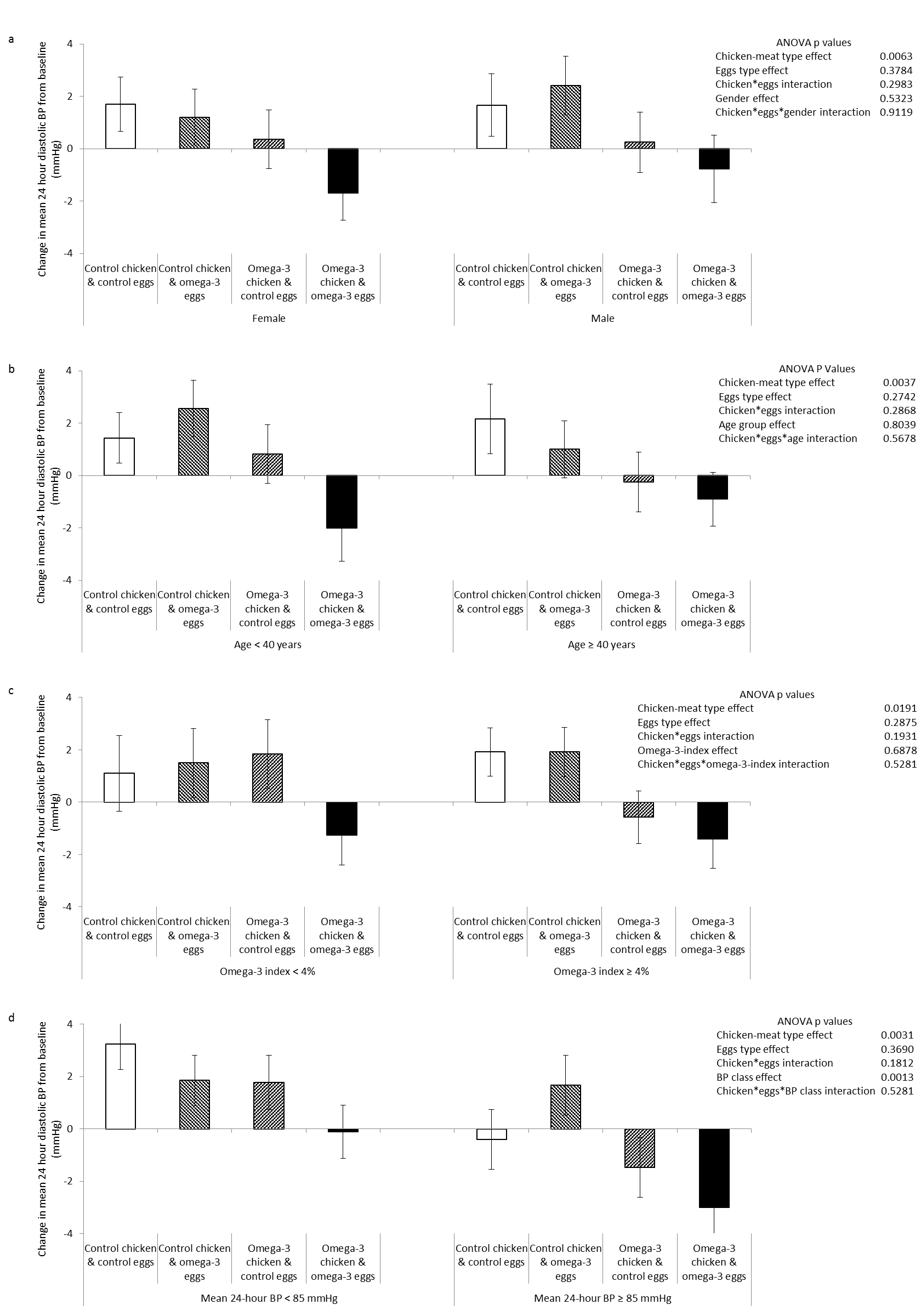
**

**Supplemental Table 1:** EPA and DHA content in control and omega-3-PUFA enriched chicken-meat and eggs

| **Food Product** | | **EPA mg/100g** | **DHA mg/100g** | **Portion size g** | **EPA mg/portion** | **DHA mg/portion** |
| --- | --- | --- | --- | --- | --- | --- |
| Eggs |  |  |  |  |  |  |
|  | Control Egg | 0 | 54 | 60 | 0 | 32 |
|  | Omega-3-PUFA Enriched Egg | 5 | 200 | 60 | 3 | 120 |
|  |  |  |  |  |  |  |
| Chicken-meat |  |  |  |  |  |  |
|  | Control Chicken Breast | 3.5 | 5 | 120 | 4 | 6 |
|  | Omega-3-PUFA Enriched Chicken Breast | 12 | 65 | 120 | 14 | 78 |
|  |  |  |  |  |  |  |
|  | Control Chicken Thigh | 10 | 11 | 120 | 12 | 13 |
|  | Omega-3-PUFA Enriched Chicken Thigh | 30 | 145 | 120 | 36 | 174 |
|  |  |  |  |  |  |  |

**Supplemental Table 2:** EPA and DHA Intakes According to Diet.

|  | | **Control Chicken & Control Eggs** | **Control Chicken & Omega-3-PUFA Eggs** | **Omega-3-PUFA Chicken & Control Eggs** | **Omega-3-PUFA Chicken & Omega-3-PUFA Eggs** |
| --- | --- | --- | --- | --- | --- |
| **Chicken portions eaten/week** | | 4.0±0.9 | 3.9±1.6 | 3.6±0.7 | 3.6±0.7 |
| **White meat %** | | 64±16 | 59±15 | 70±15 | 69±16 |
| **Dark meat %** | | 36±16 | 41±15 | 30±15 | 31±16 |
| **Eggs eaten/week** | | 5.9±2.5 | 5.0±2.3 | 5.2±1.9 | 5.1±1.8 |
| **EPA intake (mg/week)** | from white meat | 10.4±2.9 | 9.4±3.4 | 35.9±8.6 | 35.4±10.3 |
|  | from dark meat | 17.4±9.9 | 19.9±13.0 | 40.7±24.2 | 40.5±22.6 |
|  | from eggs | 0 | 15.1±7.0 | 0 | 15.2±5.5 |
|  | total from meat and eggs | 27.8±9.3 | 44.5±18.6 | 76.7±21.9 | 91.2±21.5 |
| **DHA intake (mg/week)** | from white meat | 14.9±4.1 | 13.5±4.8 | 194.7±46.7 | 191.8±55.6 |
|  | from dark meat | 19.5±11.1 | 22.4±14.6 | 196.8±117.1 | 196.0±109.3 |
|  | from eggs | 189.9±80.4 | 602.9±278.3 | 169.7±60.6 | 609.8±221.0 |
|  | total from meat and eggs | 224.3±86.3 | 638.8±285.9 | 561.3±132.0 | 997.6±255.7 |
| **Total EPA+DHA intake from chicken meat and eggs (mg/week)** | | 252.1±92.1 | 683.3±299.8 | 637.9±151.8 | 1088.9±271.5 |

Data are mean±SD.

**Supplemental Table 3:** Changes in Red Cell and Plasma Levels of EPA and DHA after Consumption of Control or Omega-3 PUFA Enriched Chicken-meat and Eggs for 3 and 6 Months.

|  | | | **Control Chicken & Control Eggs (n=42)** | **Control Chicken & Omega-3-PUFA Eggs (n=40)** | **Omega-3-PUFA Chicken & Control Eggs (n=40)** | **Omega-3-PUFA Chicken & Omega-3-PUFA Eggs (n=39)** | **ANOVA P values** | | |
| --- | --- | --- | --- | --- | --- | --- | --- | --- | --- |
|  |  |  |  |  |  |  | **Chicken-Meat Effect** | **Eggs Effect** | **Chicken* Eggs Interaction** |
| **Red Cell EPA+DHA (% of total lipids)** | Baseline | | 4.54±1.47 | 4.60±1.78 | 4.61±1.69 | 4.33±1.60 |  |  |  |
|  | 3 months | Change from baseline | -0.74 (0.25) | 0.18 (0.25) | -0.04 (0.26) | 0.72 (0.25) | 0.0149 | 0.0010 | 0.7595 |
|  |  | Difference from control foods | na | 0.92 [0.04, 1.81] ^0.0399^ | 0.70 [-0.20, 1.60] ^ns^ | 1.47 [0.57, 2.36] ^0.0003^ |  |  |  |
|  | 6 months | Change from baseline | -0.55 (0.26) | 0.34 (0.25) | 0.24 (0.28) | 1.12 (0.28) | 0.0036 | 0.0012 | 0.9878 |
|  |  | Difference from control foods | na | 0.89 [-0.02, 1.79] ^ns^ | 0.79 [-0.15, 1.74] ^ns^ | 1.67 [0.73, 2.62]^<0.0001^ |  |  |  |
| **Red Cell EPA (% of total lipids)** | Baseline | | 0.81±0.36 | 0.93±0.40 | 0.90±0.44 | 0.89±0.48 |  |  |  |
|  | 3 months | Change from baseline | -0.18 (0.06) | -0.05 (0.06) | -0.08 (0.06) | -0.04 (0.06) | 0.3664 | 0.1399 | 0.4727 |
|  |  | Difference from control foods | na | 0.13 [-0.08, 0.33] ^ns^ | 0.09 [-0.11, 0.30] ^ns^ | 0.14 [-0.07, 0.35] ^ns^ |  |  |  |
|  | 6 months | Change from baseline | -0.15 (0.06) | -0.09 (0.06) | 0.01 (0.06) | 0.01 (0.06) | 0.0342 | 0.5801 | 0.5796 |
|  |  | Difference from control foods | na | 0.07 [-0.13, 0.27] ^ns^ | 0.16 [-0.05, 0.37] ^ns^ | 0.16 [-0.05, 0.37] ^ns^ |  |  |  |
| **Red Cell DHA (% of total lipids)** | Baseline | | 3.73±1.19 | 3.67±1.44 | 3.70±1.35 | 3.44±1.20 |  |  |  |
|  | 3 months | Change from baseline | -0.56 (0.21) | 0.23 (0.20) | 0.04 (0.21) | 0.77 (0.21) | 0.0065 | 0.0003 | 0.8636 |
|  |  | Difference from control foods | na | 0.79 [0.07, 1.51] ^0.0264^ | 0.60 [-0.13, 1.34] ^ns^ | 1.33 [0.60, 2.06]^<0.0001^ |  |  |  |
|  | 6 months | Change from baseline | -0.40 (0.21) | 0.42 (0.21) | 0.24 (0.23) | 1.11 (0.23) | 0.0030 | 0.0002 | 0.8950 |
|  |  | Difference from control foods | na | 0.82 [0.07, 1.57] ^0.0270^ | 0.63 [-0.15, 1.41] ^ns^ | 1.51 [0.73, 2.29]^<0.0001^ |  |  |  |

**Supplemental Table 3:** Changes in Red Cell and Plasma Levels of EPA and DHA after Consumption of Control or Omega-3 PUFA Enriched Chicken-meat and Eggs for 3 and 6 Months. (Continued)

|  | | | **Control Chicken & Control Eggs (n=42)** | **Control Chicken & Omega-3-PUFA Eggs (n=40)** | **Omega-3-PUFA Chicken & Control Eggs (n=40)** | **Omega-3-PUFA Chicken & Omega-3-PUFA Eggs (n=39)** | **ANOVA P values** | | |
| --- | --- | --- | --- | --- | --- | --- | --- | --- | --- |
|  |  |  |  |  |  |  | **Chicken-Meat Effect** | **Eggs Effect** | **Chicken* Eggs Interaction** |
| **Plasma EPA+DHA (ug/g)** | Baseline | | 69.0±25.1 | 83.1±32.9 | 81.1±35.6 | 79.3±29.9 |  |  |  |
|  | 3 months | Change from baseline | -1.3 (4.3) | 3.1 (4.3) | 9.5 (4.4) | 5.9 (4.4) | 0.1175 | 0.9262 | 0.3597 |
|  |  | Difference from control foods | na | 4.4 [-10.8, 19.6] ^ns^ | 10.9 [-4.5, 26.2] ^ns^ | 7.3 [-8.2, 22.7] ^ns^ |  |  |  |
|  | 6 months | Change from baseline | -1.3 (4.9) | 0.4 (4.8) | 11.1 (5.1) | 12.3 (5.1) | 0.0156 | 0.7746 | 0.9581 |
|  |  | Difference from control foods | na | 1.7 [-15.4, 18.6] ^ns^ | 12.4 [-5.1, 30.0] ^ns^ | 13.6 [-4.1, 31.3] ^ns^ |  |  |  |
| **Plasma EPA (ug/g)** | Baseline | | 22.2±10.4 | 29.3±16.0 | 27.8±17.0 | 28.3±15.8 |  |  |  |
|  | 3 months | Change from baseline | -1.3 (2.5) | -0.6 (2.5) | 2.4 (2.6) | -3.3 (2.6) | 0.8608 | 0.3249 | 0.2117 |
|  |  | Difference from control foods | na | 0.7 [-8.3, 9.7] ^ns^ | 3.7 [-5.4, 12.7] ^ns^ | -2.1 [-11.2, 7.0] ^ns^ |  |  |  |
|  | 6 months | Change from baseline | -1.6 (2.6) | -3.1 (2.6) | 3.4 (2.7) | -0.5 (2.7) | 0.1530 | 0.3109 | 0.6518 |
|  |  | Difference from control foods | na | -1.5 [-10.6, 7.6] ^ns^ | 5.0 [-4.3, 14.3] ^ns^ | 1.1 [-8.3, 10.5] ^ns^ |  |  |  |
| **Plasma DHA (ug/g)** | Baseline | | 46.8±16.3 | 53.7±20.7 | 53.2±21.4 | 50.9±15.8 |  |  |  |
|  | 3 months | Change from baseline | -0.1 (2.2) | 3.7 (2.2) | 7.1 (2.3) | 9.2 (2.3) | 0.0050 | 0.1925 | 0.7285 |
|  |  | Difference from control foods | na | 3.7 [-4.1, 11.6] ^ns^ | 7.2 [-0.7, 15.1] ^ns^ | 9.3 [1.4, 17.3] ^0.0156^ |  |  |  |
|  | 6 months | Change from baseline | 0.3 (2.8) | 3.5 (2.8) | 7.7 (3.0) | 12.8 (3.0) | 0.0045 | 0.1581 | 0.7478 |
|  |  | Difference from control foods | na | 3.2 [-6.9, 13.1] ^ns^ | 7.4 [-2.8, 17.7] ^ns^ | 12.5 [2.2, 22.8] ^0.0120^ |  |  |  |

Baseline values are mean±SD. Changes from baseline are mean change (SEM). Differences from control foods are mean difference [98.75 % Confidence Intervals]^Bonferroni adjusted P value^.

**Supplementary Table 4:** Changes in Mean 24-hour Ambulatory Blood Pressure and Heart Rate after Consumption of Control or Omega-3 PUFA Enriched Chicken-meat and Eggs for 3 and 6 Months.

|  | | | **Control Chicken & Control Eggs (n=42)** | **Control Chicken & Omega-3-PUFA Eggs (n=40)** | **Omega-3-PUFA Chicken & Control Eggs (n=40)** | **Omega-3-PUFA Chicken & Omega-3-PUFA Eggs (n=39)** | **ANOVA P values** | | |
| --- | --- | --- | --- | --- | --- | --- | --- | --- | --- |
|  |  |  |  |  |  |  | **Chicken-Meat Effect** | **Eggs Effect** | **Chicken* Eggs Interaction** |
| **Mean 24-hour Systolic BP (mmHg)** | Baseline | | 116.4±6.9 | 116.1±8.2 | 116.0±9.0 | 115.2±9.9 |  |  |  |
|  | 3 months | Change from baseline | -0.5 (1.0) | -0.2 (1.0) | -1.5 (1.0) | -0.2 (1.0) | 0.5994 | 0.4211 | 0.5701 |
|  |  | Difference from control foods | na | 0.2 [-3.1, 3.6] ^ns^ | -1.1 [-4.5, 2.4] ^ns^ | 0.3 [-3.2, 3.7] ^ns^ |  |  |  |
|  | 6 months | Change from baseline | 0.6 (1.0) | 0.9 (1.0) | 0.0 (1.0) | -1.2 (1.0) | 0.1770 | 0.6489 | 0.4606 |
|  |  | Difference from control foods | na | 0.3 [-3.1, 3.7] ^ns^ | -0.6 [-4.1, 2.9] ^ns^ | -1.8 [-5.3, 1.7] ^ns^ |  |  |  |
| **Mean 24-hour Diastolic BP (mmHg)** | Baseline | | 68.9±7.0 | 67.7±6.2 | 69.3±6.8 | 68.4±5.7 |  |  |  |
|  | 3 months | Change from baseline | 0.6 (0.8) | 0.6 (0.8) | -0.2 (0.8) | -0.3 (0.8) | 0.2880 | 0.9005 | 0.9270 |
|  |  | Difference from control foods | na | 0.0 [-2.7, 2.7] ^ns^ | -0.7 [-3.4, 2.0] ^ns^ | -0.9 [-3.6, 1.8] ^ns^ |  |  |  |
|  | 6 months | Change from baseline | 1.7 (0.8) | 1.7 (0.7) | 0.2 (0.8) | -1.3 (0.8) | 0.0035 | 0.3094 | 0.3048 |
|  |  | Difference from control foods | na | 0.0 [-2.6, 2.7] ^ns^ | -1.5 [-4.2, 1.2] ^ns^ | -3.1 [-5.8, -0.3] ^0.0231^ |  |  |  |
| **Mean 24-hour Heart Rate (beats /minute)** | Baseline | | 69.5±8.0 | 66.4±9.3 | 68.0±9.0 | 69.2±10.5 |  |  |  |
|  | 3 months | Change from baseline | 2.1 (1.0) | 0.4 (1.0) | 0.3 (1.0) | 1.5 (1.0) | 0.7199 | 0.8092 | 0.1597 |
|  |  | Difference from control foods | na | -1.7 [-5.3, 1.9] ^ns^ | -1.8 [-5.4, 1.8] ^ns^ | -0.6 [-4.2, 3.0] ^ns^ |  |  |  |
|  | 6 months | Change from baseline | 2.2 (1.0) | 0.9 (1.0) | -1.8 (1.0) | 0.1 (1.0) | 0.0226 | 0.8107 | 0.1290 |
|  |  | Difference from control foods | na | -1.3 [-4.9, 2.2] ^ns^ | -4.0 [-7.6, -0.3] ^0.0302^ | -2.1 [-5.8, 1.5] ^ns^ |  |  |  |

Baseline values are mean±SD. Changes from baseline are mean change (SEM). Differences from control foods are mean difference [98.75 % Confidence Intervals]^Bonferroni adjusted P value^.
